# Supplementary material for: Nonlinear feedforward enabling quantum computation
Source: Nat Commun. 2023 Jul 12;14:3817. doi: 10.1038/s41467-023-39195-w (PMC10338683; doi:10.1038/s41467-023-39195-w)
Supplement: Supplementary file 1 — Supplementary Information [file 41467_2023_39195_MOESM1_ESM.pdf]

Supplementary Information

Supplementary Note 1 - Calibration of input states

For calibrating the input coherent state, we utilize the experimental setup as a heterodyne measurement. The heterodyne measurement of the input state is simply done if we observe the outcomes of two homodyne detectors at $100\,\mathrm{ns}$ before the arrival of non-Gaussian ancillary state. At this timing, the ancillary state can be regarded as a vacuum state since we pump the OPO in weak pump condition, as well as the feedforward is deactivated to set the measured basis of two homodyne detectors to $\hat{x}$ and $\hat{p}$. Since the coherent state rotates at $5.5\,\mathrm{MHz}$ in the phase space, the exact input state can be estimated from the measurement results. We measure the shot noise of the homodyne detectors, which is used to calibrate the electrical outcomes, blocking the input states and ancillary states.

As mentioned in the main text, we choose the amplitude from 0.0 to 3.5 by 27 steps, which is a range where the feedforward circuit is not saturated. For each amplitude, 80000 frames (including two measurement outcomes of homodyne detectors and a phase reference signal for the input coherent states) are recorded by an oscilloscope. We fit the measured outcomes in each amplitudes to a complex amplitudes, $f(A, \phi_{\text{offset}}) = A \exp\left[i\left(-\phi + \phi_{\text{offset}}\right)\right] + (x_0 + ip_0)$, where A is the amplitude, ϕ is the phase of reference signal, ϕ_{offset} is a phase offset from the reference, x_0 and p_0 are offset of x and p quadratures. A, ϕ_{offset}, x_0, and y_0 are the fitting parameters.

Supplementary Figure 1 shows the distribution of fitted coherent states. The fluctuation of two quadrature offsets are negligibly small compared to the amplitudes of coherent states. The amplitudes are stepped equally in enough fine resolution since the distributions of the detector states are derived from the ancillary states (see Eq.(19)), which has no steep structures in the phase space. The phases of the input coherent states are randomized uniformly as intended.

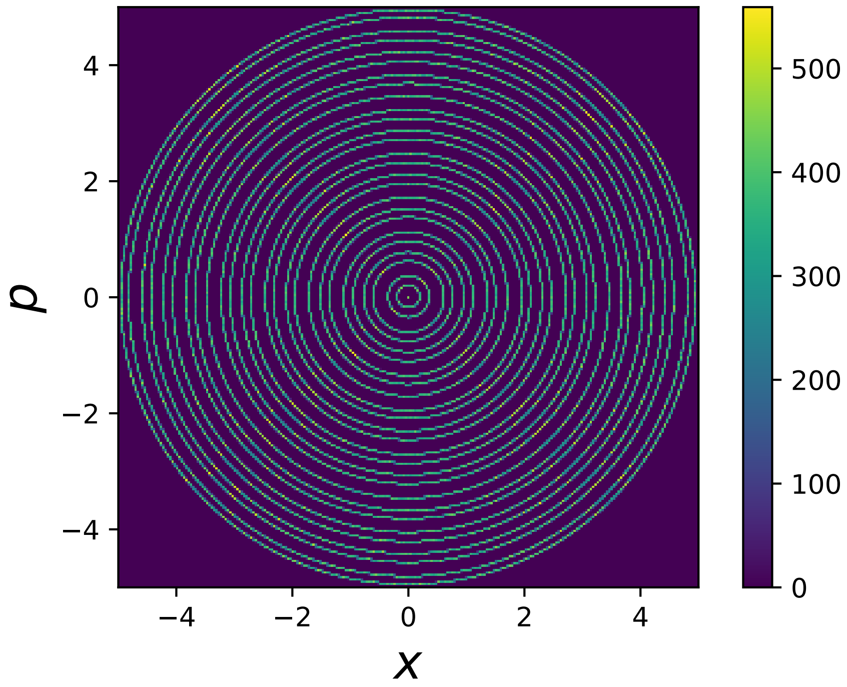

Supplementary Figure 1: **Distribution of coherent input states.** The distribution of input coherent state is shown in a two-dimensional histogram. The brightness of each amplitude is corrected by multiplying its amplitude, since the number of data points in each bin is inverse proportional to the amplitude and hard to see the region of large amplitude. The color bar shows the corrected density of the input states and the values are not normalized.

Supplementary Note 2 - Post-processing for the measurement outcomes

After two measurement outcomes are obtained from two homodyne detectors, we correct the effect of residual coherent state before we apply nonlinear gain $g(q)$. This is because coherent state is injected continuously at a single frequency, while the measurement system works for a specific wave packet. We perform the nonlinear feedforward to the second homodyne detector by a few nanoseconds before the arrival of the non-Gaussian ancillary state. However, the impulse response of our measurement device has a long tail in time-domain because of a high-pass filter with a cuf-off frequency of $100\,\mathrm{kHz}$ which is contained in the homodyne detectors to remove noisy low-frequency components from their electrical outcomes. The filter has little effect when we consider the case of the non-Gaussian ancilla localized in time, because this effect is as the same as negligible loss. When we consider the case of continuous-wave input, however, the outcomes of HD2 include information from different phase coherent states before feedforward. Thus, the effect is more significant if the amplitude of coherent state is large.

The model is explained as follows. We define a real temporal mode function $f(t)$, which is the same mode function as the ancillary state. To consider the phase rotation by the feedforward, we consider a complex temporal mode $f(t)e^{i\theta(t)}$ where $\theta(t)$ is a rotated phase. If we assume the nonlinear feedforward instantly rotates the phase of the measurement basis, $\theta(t)$ is a step function,

$$\theta(t) = \begin{cases} 0 & (t < t_\mathrm{f}) \\ \Theta & (t_\mathrm{f} \leq t) \end{cases} \tag{1}$$

where t_f is the trigger timing of the nonlinear feedforward and Θ is the rotated angle. The contribution of coherent states in the measured value is

$$\int_{-\infty}^{t_\mathrm{f}} f(t)|\alpha| \sin\left(\Omega t + \phi\right) \mathrm{d}t + \int_{t_\mathrm{f}}^{\infty} f(t)|\alpha| \sin\left(\Omega t + \phi + \Theta\right) \mathrm{d}t \tag{2}$$

where Ω is angular frequency of the coherent state and ϕ is the phase offset of the coherent state. Because the mode function $f(t)$ is localized around $t = 0$ even with the long tail, if the

timing of nonlinear feedforward operation is sufficiently earlier than the arrival of wave packet, in other words $t_F \to -\infty$, this contribution becomes

$$\int_{-\infty}^{\infty} f(t)|\alpha| \sin{(\mathit{\Omega} t + \phi + \mathit{\Theta})}\, \mathrm{d}t \tag{3}$$

This is what we should measure. Thus, the residual offset is calculated as

$$c(\phi, \mathit{\Theta}) = |\alpha| \int_{-\infty}^{t_\mathrm{f}} f(t) \left[\sin{(\mathit{\Omega} t + \phi + \mathit{\Theta})} - \sin{(\mathit{\Omega} t + \phi)}\right] \mathrm{d}t \tag{4}$$

We experimentally characterize the correction factor $c(\phi, \mathit{\Theta})$. We input coherent states and vacuum ancillay states to the experimental setup and program the LUT to rotate the phase of the local oscillator $\mathit{\Theta}$ by +90 and -90 degrees. The measured value is actually Eq.(2) but the second term can be cancelled by summing the two results with +90 and -90 degrees. The estimated the correction factor is $c(\phi, \mathit{\Theta}) = 0.161|\alpha| \left[\sin(\mathit{\Omega} t + \phi + \mathit{\Theta} - 0.812) - \sin(\mathit{\Omega} t + \phi - \mathit{0.812})\right]$.

Note that, to avoid this correction, preparation of coherent states in a localized wave packet is possible in principle, but it requires much longer optical delay lines to wait the preparation of the localized input states after the heralding events because the heralding signals appear at random timings. After the correction, the outcomes are multiplied with the nonlinear gain $g(q) = \sqrt{2}/\cos\theta(q) = \sqrt{1 + 2\gamma^2 q^2}$ to obtain the measurement result of whole setup.

Supplementary Note 3 - First and second moments of measured nonlinear quadratures

The nonlinear feedforward enables us to access the nonlinear quadrature of the input states, including a nonlinear quadrature term of ancillary state as followed:

$$\hat{m} = \hat{p}_{\mathrm{in}} + \gamma \hat{x}_{\mathrm{in}}^2 + \left(\hat{p}_{\mathrm{anc}} - \gamma \hat{x}_{\mathrm{anc}}^2\right) \tag{5}$$

As a simple check, we calculate first and second moments of the measurement outcomes.

Supplementary Figure 2 shows the statistics of the measurement outcomes m as a function of the input coherent states. We observe the measurement outcomes m depend quadratically on

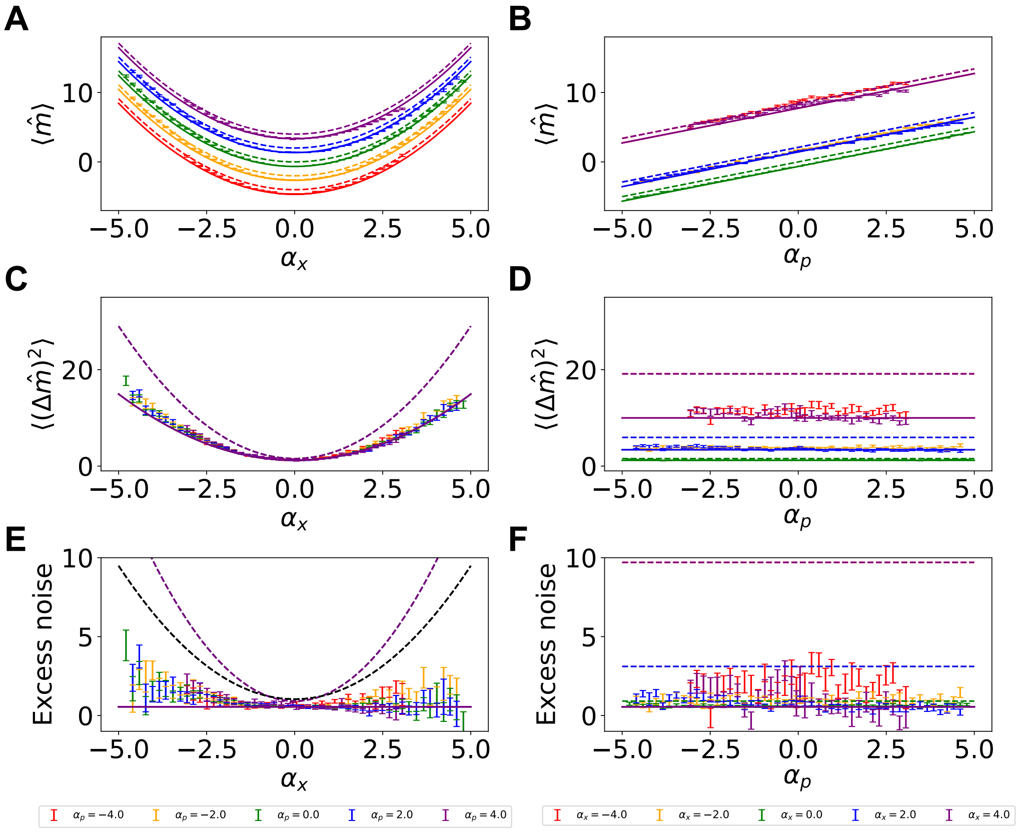

Supplementary Figure 2: **Outcomes of the tailored measurement versus amplitudes of input coherent states.** (A) Mean values of the measurement outcomes m as a function of α_x with fixed α_p. (B) Mean values of the measurement outcomes m as a function of α_p with fixed α_x. (C) and (D) show the variance of the measurement outcomes m as a function of α_x and α_p. (E) and (F) show the excess noise of the measurement outcomes m as a function of α_x and α_p. Solid lines show the theoretical predictions calculated with the measured ancillary state. Dashed lines are theoretical plots without the nonlinear feedforward. The errorbars are unbiased standard deviations for each vertical axis calculated by bootstrapping method.

α_x (Suppl.Fig.2A and 2C), and linearly on α_p (Suppl.Fig.2B and 2D). The experimental mean values (Suppl.Fig.2A and 2B) also show good agreement with the theoretical predictions based on the ancillary state used in the experiment. On the other hand, the experimental variances (Suppl.Fig.2C and 2D) agree with the theoretical predictions with small α_x, while show a relatively large deviation when α_x is large. This is because the accuracy of arctangent calculations in the nonlinear feedforward is limited for the larger α_x in addition to less number of data for larger α_x with $\alpha_p = 0$. The deviation of the variances does not depend on α_p since the value is not measured by the homodyne measurement HD1 and not used in the nonlinear feedforward.

Dashed lines in Suppl.Fig.2 represent theoretical lines without feedforward, highlighting the importance of the nonlinear feedforward. Without feedforward, the system turns out to be an non-adaptive linear heterodyne measurement[1], where $\hat{x}_{\mathrm{in}}$ and $\hat{p}_{\mathrm{in}}$ are simultaneously measured with quantum noise of the ancillary state, $\hat{x}_{\mathrm{anc}}$ and $\hat{p}_{\mathrm{anc}}$. If the measured values of the heterodyne measurement, q', p' are nonlinearly processed to calculate $\sqrt{2}p' + 2\gamma q'^2$, the processed measurement result $\hat{m}_{\mathrm{het}}$ is given by

$$\hat{m}_{\mathrm{het}} = \hat{p}_{\mathrm{in}} + \gamma\hat{x}_{\mathrm{in}}^2 + \left(\hat{p}_{\mathrm{anc}} + \gamma\hat{x}_{\mathrm{anc}}^2\right) - 2\hat{x}_{\mathrm{in}}\hat{x}_{\mathrm{anc}} \tag{6}$$

This is a kind of measurement about $\hat{p}_{\mathrm{in}} + \gamma\hat{x}_{\mathrm{in}}^2$. In classical schemes, this setup works well since we can ignore the noise from the ancillary states. Compared to the unbiased noise terms in Eq.(5), however, the last term of Eq. (6) indicates that the noise term is biased by $\hat{x}_{\mathrm{in}}$ of the input, and cannot be cancelled by any ancillary states. (Eigenstates of $\hat{x}$ suppress the cross term, but $\hat{p}$ of the states completely cover the measurement results of the input states.) Note that we use the vacuum state as the ancillary state for the dashed lines in Suppl.Fig.2. Supplementary Figure 2C and 2D show that our nonlinear feedforward eliminates this unwanted dependence of noise on x quadratures of the input. Although the variance of experimental data is not completely unbiased to the input state due to the imperfection in the experiment, the additional noise is less

biased and reduced by at least 40% from the case without feedforward.

Moreover, the advantage of our measurement over inadaptive Gaussian measurements is verified by the excess noise (Suppl.Fig.2E and 2F). A general inadaptive Gaussian measurement can be simplified to an unbalanced heterodyne measurement without the nonlinear feedforward and it has a biased noise term when used for the measurement of $\hat{p}_{\rm in} + \gamma\hat{x}_{\rm in}^2$ by a nonlinear post-processing. This noise term can be minimized with respect to a known set of input coherent states but it can never be completely removed. Black dashed line shows the case of optimal Gaussian measurement minimizing the average excess noise for coherent states of the same distribution of the experimental input states. The excess noise of our nonlinear quadrature measurement is smaller than the bound of nonlinearly processed Gaussian measurements without nonlinear feedforward in all input states. Therefore, our nonlinear quadrature measurement overcomes general Gaussian measurements via a non-Gaussianity induced by the nonlinear feedforward.

Supplementary Note 4 - Detector tomography of nonlinear quadrature measurement

For evaluation of quantum property of our measurement, we perform detector tomography of the tailored measurement and reconstruct the detector states (POVM elements) via an iterative maximum likelihood method [2]. In the analysis, we limit the area of POVM elements in the phase space representation because we can correctly reconstruct the POVM elements only within the area covered by coherent probe states.

To confirm the area occupied by the POVM elements, we theoretically calculate the POVM elements predicted from the measured ancillary state (see Eq.(20)). Supplementary Figure 3 shows the Wigner functions of predicted POVM elements with different integral ranges of q and different m. Note that the each POVM element is renormalized as the trace to be 1. The

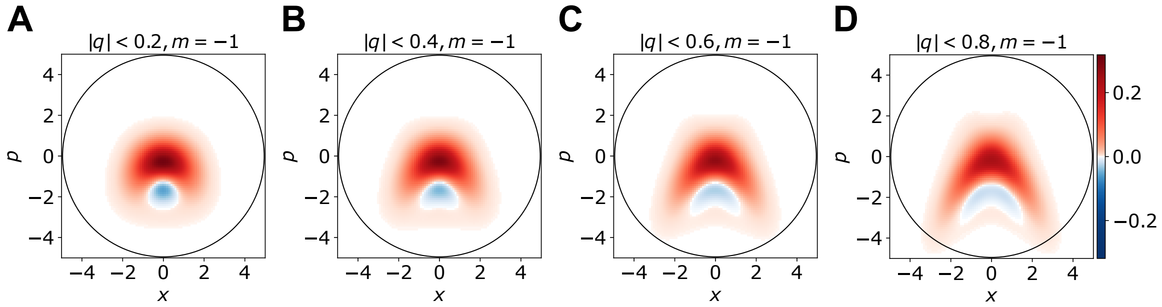

Supplementary Figure 3: **Theoretical POVM elements with different integral ranges.** Wigner functions of ideal detector states associated with $m = -1$. Note that the ancillary state used for the plot is a pure state, $|\psi\rangle = 0.8\,|0\rangle + 0.6i\,|1\rangle$. The range of q is (A) $|q| < 0.2$, (B) $|q| < 0.4$, (C) $|q| < 0.6$, and (D) $|q| < 0.8$. Black circle shows the area covered by the input states.

POVM elements corresponding to $-1 \le m \le 1$ and $-0.6 \le q \le 0.6$ is almost inside the area scanned by the input states.

These boundaries of integral ranges are double-checked via a distribution of measurement outcomes with input coherent states on the boundary of the covered area. The probability to obtain a certain measurement outcomes Q, M is calculated as

$$\mathrm{prob}(Q, M|\alpha) = \mathrm{Tr}\left[|\alpha\rangle\langle\alpha|\,\hat{\Pi}_{Q,M}\right] = \iint W_\alpha(x,p) W_{\hat{\Pi}_{Q,M}}(x,p)\mathrm{d}x\mathrm{d}p \tag{7}$$

Hence, if a POVM element which is inside the area covered by the coherent probe states, and if the coherent states of the maximum amplitude are injected, the probability to obtain the measurement outcomes regarding to the POVM element will be negligibly small. Supplementary Figure 4 shows the minimal range r where $|q| < r$ with given m include only N events when $|\alpha| = 3.5$. Within $|q| < 0.6$ and $-1 \le m \le 1$, almost no event is observed on the boundary input states. Note that if we choose $|\alpha| < 3.5$, we have about 240,000 events within $|q| < 0.6$ and $-1 \le m \le 1$ in total. The measurement outcomes m are distributed continuously, but for the sake of evaluation we discretize the measurement range into 20 events and reconstruct the respective detector states.

Supplementary Figure 5 shows the Wigner functions of reconstructed detector states asso-

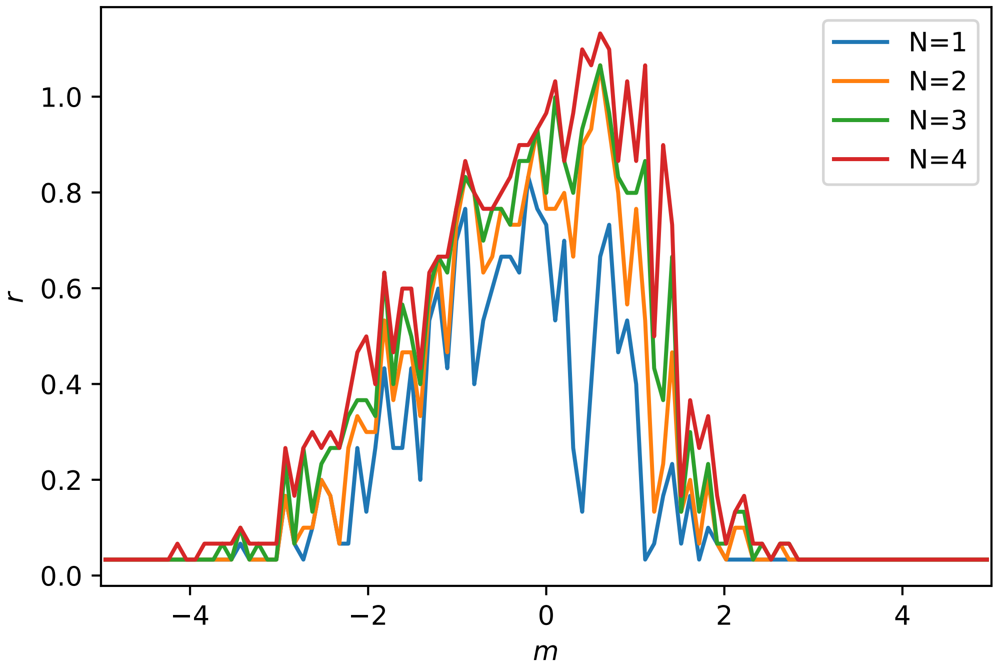

Supplementary Figure 4: **Safety range of q for a given m.** The plot shows the minimal range of q ($|q| < r$) for the given m including only N events on the boundary coherent states.

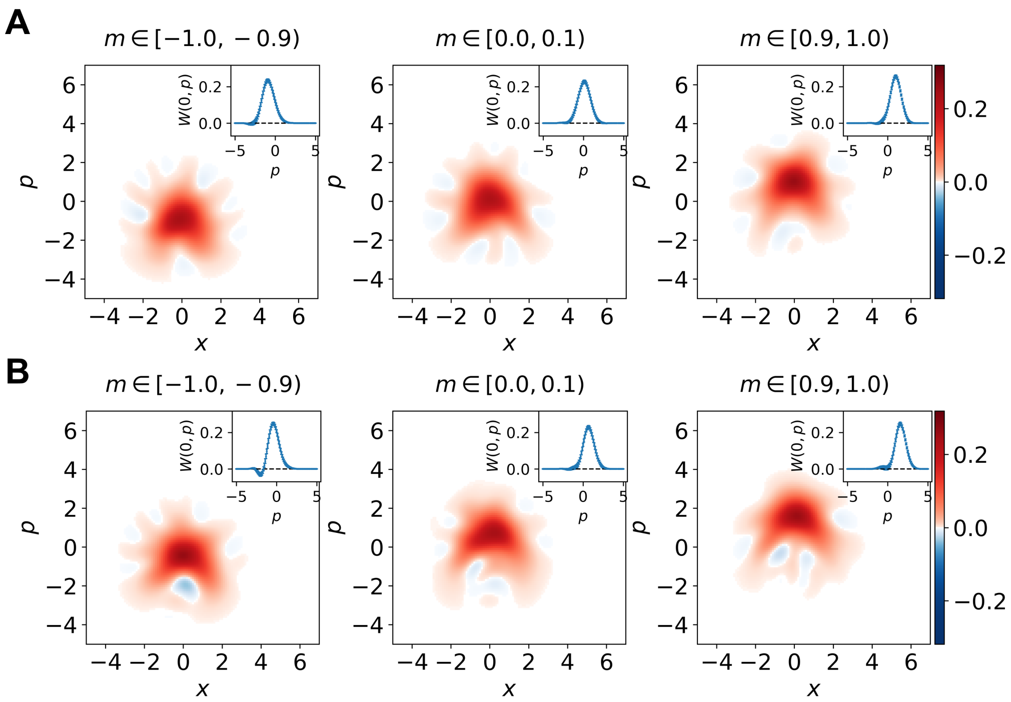

Supplementary Figure 5: **Wigner functions of reconstructed detector states.** Wigner functions of the reconstructed detector states associated with the outcome m in $[-1.0, -0.9)$, $[0.0, 0.1)$, and $[0.9, 1.0)$ injecting (A) a vacuum ancillary state, and (B) the non-Gaussian ancillary state. The insets show the cross section of Wigner functions along $x = 0$ with error bars calculated by a bootstrapping method.

ciated with different measurement outcomes. The detector states are displaced in p-direction by the measurement outcomes. Though Wigner functions have some ripples, the detector states have more sharp parabolic shapes with the non-Gaussian ancillary states compared to vacuum ancillary states.

Supplementary Note 5 - Artifacts in the reconstruction method

The area with negative values in the Wigner functions of the detector states should be induced by a negativity in the quantum non-Gaussian ancillary states, that is also induced via a two-mode entanglement and on/off detection on the idler mode. Even in the case of vacuum ancillary states, however, there are some ripples around the Wigner functions with negative values.

The ripples are considered as artifacts in the reconstruction method, regarding finite photon number subspace and finite number of experimental results. Supplementary Figure 6 shows the effect of these configurations in the reconstruction method. The ripples are emphasized with more maximum photon numbers calculated in the reconstruction method. More data points reduce the artifacts but still visible artifacts remain. Note that more data points do not necessarily have advantage in actual experiment, because much longer time is required to obtain 10 times larger number of data and the entire system became more unstable.

Supplementary Note 6 - Noise reduction by the non-Gaussian ancillary state

The non-Gaussian ancillary state lets the detector states be nearer to that of ideal nonlinear quadrature measurement of $\hat{p} + \gamma\hat{x}^2$. The detector states of the ideal nonlinear quadrature measurement are p-displaced cubic phase state (CPS), in other words, eigenstates of $\hat{p} + \gamma\hat{x}^2$. As discussed in [3], the variance of $\hat{p} + \gamma\hat{x}^2$ is an indicator of the similarity to the displaced CPS. Hence, the advantage of the non-Gaussian ancillary state is quantitively characterized as the reduction of the variances of $\hat{p} + \gamma\hat{x}^2$ of the normalized POVM elements, $\hat{\Pi}_m/\mathrm{Tr}\left[\hat{\Pi}_m\right]$.

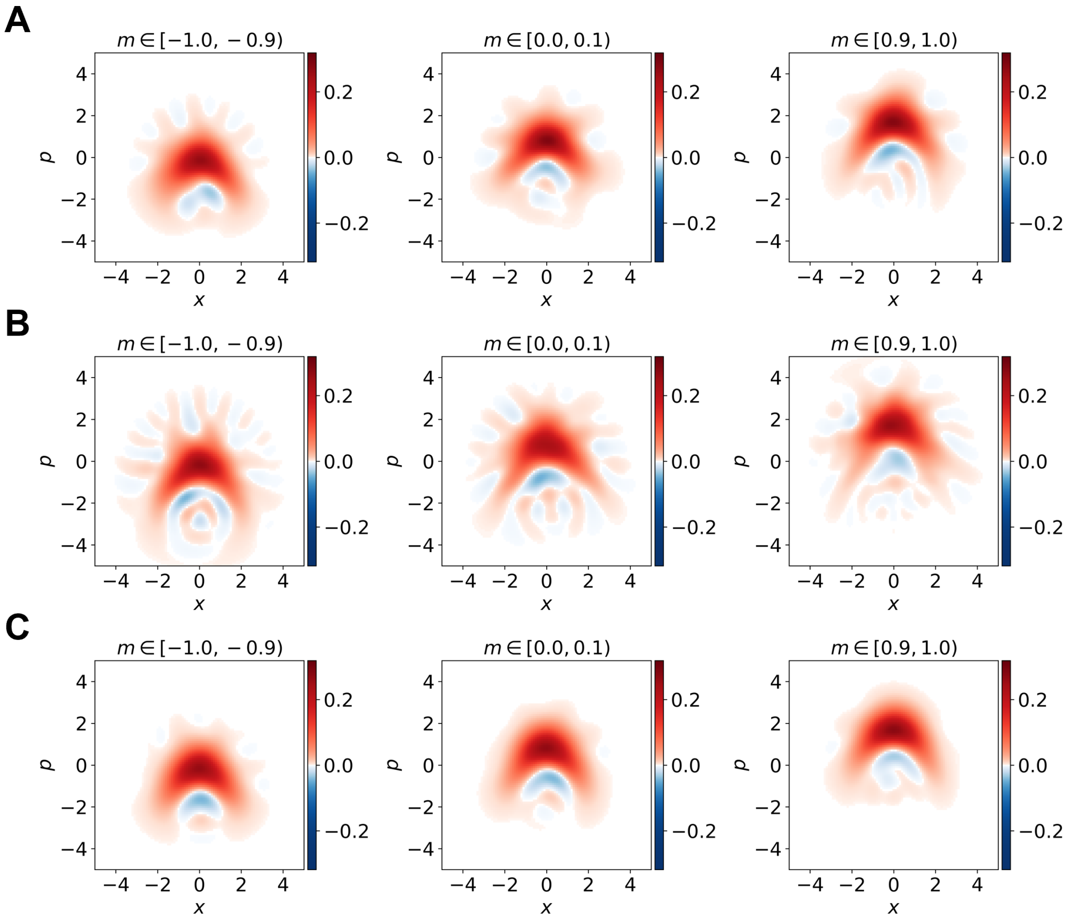

Supplementary Figure 6: **Detector states reconstructed from Monte-Carlo simulations with different configuration.** Wigner functions of detector states are reconstructed with different maximum photon number $N_{\rm max}$ and different number of data points. (A) $N_{\rm max} = 10$ with 2.16 million points, (B) $N_{\rm max} = 15$ with 2.16 million points, and (C) $N_{\rm max} = 10$ with 21.6 million points. The data points are theoretically generated via Monte-Carlo simulation based on Supplementary Note 8, with $\eta_1 = \eta_2 = 1$, and pure non-Gaussian ancilla $\hat{\rho}_{\rm A} = |\psi\rangle\langle\psi|$, $|\psi\rangle = 0.8\,|0\rangle - 0.6i\,|1\rangle$.

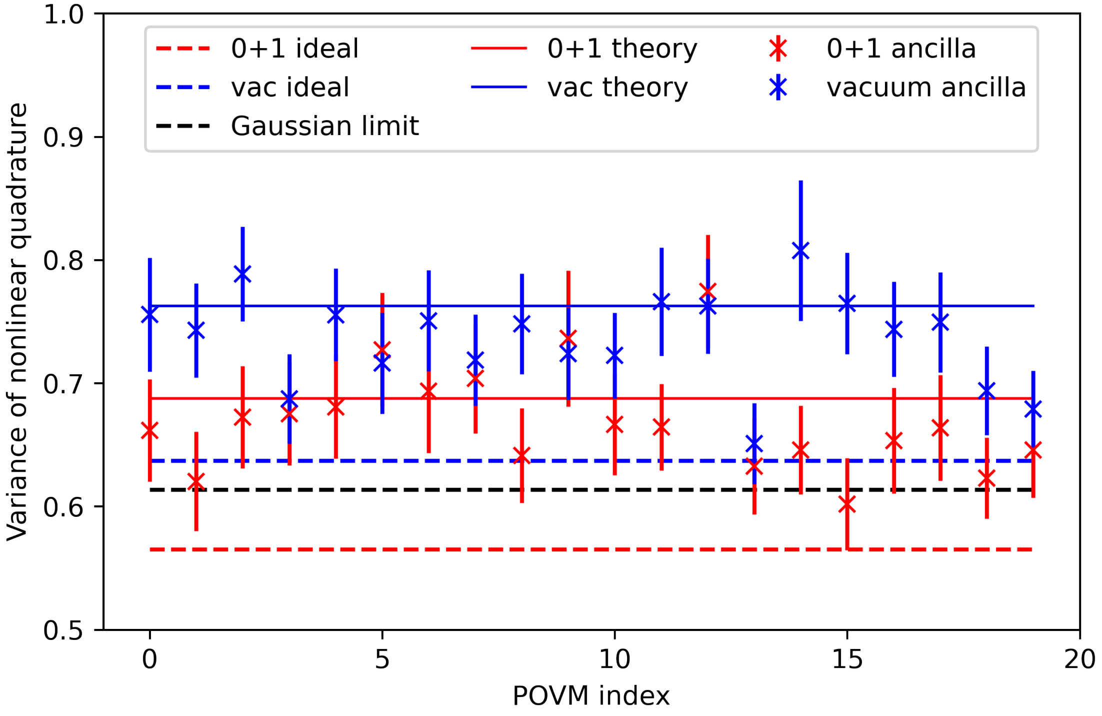

Supplementary Figure 7: **Excess noise level of detector states.** $\mathrm{var}(\hat{p}+\gamma\hat{x}^2)$ of experimental detector states with different measurement results. The indexes linearly correspond the ranges of measurement outcomes m, where the index number 0 means the interval $[-1.0, -0.9)$, and the index number 19 means the interval $[0.9, 1.0)$. The index 20 (events collecting the result out of bounds) is omitted. Red and blue dashed lines show ideal values of $\mathrm{var}(\hat{p}+\gamma\hat{x}^2)$, which are equal to $\mathrm{var}(\hat{p}-\gamma\hat{x}^2)$ of the non-Gaussian ancillary states and the vacuum ancillary states. Black dashed line shows the lower bound of $\mathrm{var}(\hat{p}+\gamma\hat{x}^2)$ for arbitrary Gaussian states and their mixture. Red and blue solid lines show the expected values of $\mathrm{var}(\hat{p}+\gamma\hat{x}^2)$ with actual detection efficiencies of the two homodyne detectors, $\eta_1 = 0.97$ and $\eta_2 = 0.91$. The error bars show the standard errors of each variance obtained by a bootstrapping method with 1000 times resampling.

Supplementary Figure 7 shows the variances of $\hat{p}+\gamma\hat{x}^2$ of the reconstructed detector states. The variance, 0.74 ± 0.01 with vacuum ancillary state in average, is decreased to 0.67 ± 0.01 with the non-Gaussian ancillary state, which is consistent with the expected values from the measured ancillary state, from 0.64 to 0.56. The variance is larger than the ideal case, mainly due to the measurement efficiency in the experimental setups. Depending on the numerical simulation (see Supplementary Note 8), actual experimental parameter $\eta_1 = 0.97$ and $\eta_2 = 0.91$ are consistent with the experimental results.

Supplementary Note 7 - Theory of nonlinear quadrature measurement (Schrödinger picture)

In this section, we derive a form of POVM elements of our nonlinear quadrature measurement and show the quantum non-Gaussianity of the POVM elements. The theory is based on [4], where generalized heterodyne measurement is analyzed.

First, we define basic components. Quadrature operators, $\hat{x}$ and $\hat{p}$, which are canonical conjugate and satisfy $[\hat{x},\hat{p}] = i\hbar$, correspond to real part and imaginary part of complex electric field for a temporal mode. Definition of quadrature operators has a freedom of phase θ. $\hat{x}_\theta, \hat{p}_\theta$ are defined as followed,

$$\begin{pmatrix} \hat{x}_\theta \\ \hat{p}_\theta \end{pmatrix} = \begin{pmatrix} \cos\theta & -\sin\theta \\ \sin\theta & \cos\theta \end{pmatrix} \begin{pmatrix} \hat{x} \\ \hat{p} \end{pmatrix} \tag{8}$$

Eigenstates of a quadrature operator $|x;\hat{x}\rangle$, which satisfies $\hat{x}\,|x;\hat{x}\rangle = x\,|x;\hat{x}\rangle$, can be defined theoretically. (In general, eigenstates of an operator $\hat{A}$ belonging to an eigenvalue a is described as $|a;\hat{A}\rangle$). Since homodyne measurements are measurement of a quadrature in a specific phase, they project quantum states to the eigenstates of the quadrature operator. Hence, a detector state (POVM element) of the homodyne measurement associated to the outcome q at a phase θ is,

$$\hat{\Pi}_q^{(\theta)} = |q;\hat{x}_\theta\rangle\,\langle q;\hat{x}_\theta| \tag{9}$$

In heterodyne measurement, which is also called as dual homodyne detection, the measured state is interfered with an ancillary state $\hat{\rho}_{\mathrm{anc}}$ at a balanced beamsplitter, then measured by two homodyne detectors whose measurement bases are set to orthogonal quadrature phases. Probability to obtain two outcomes q, y from the two homodyne detectors with the bases of $\hat{x}$ and $\hat{p}$ is expressed with a POVM element $\hat{\Pi}_{q,y}^{(\mathrm{het})}$,

$$\mathrm{prob}\,(q,y) = \mathrm{Tr}_{\mathrm{in}}\left[\hat{\rho}_{\mathrm{in}}\hat{\Pi}_{q,y}^{(\mathrm{het})}\right] \tag{10}$$

$$= \mathrm{Tr}\left[\hat{B}\left(\hat{\rho}_{\mathrm{in}}\otimes\hat{\rho}_{\mathrm{anc}}\right)\hat{B}^{\dagger}\left|q;\hat{x}\right\rangle\left\langle q;\hat{x}\right|\otimes\left|y;\hat{p}\right\rangle\left\langle y;\hat{p}\right|\right] \tag{11}$$

where $\hat{B}$ is an operator of the balanced beam splitter. Thus, when the ancillary state is a vacuum state, the POVM element is

$$\hat{\Pi}_{q,y}^{(\mathrm{het})} = \frac{1}{\pi\hbar}\left|\psi^{(\mathrm{het})}\left(q,y\right)\right\rangle\left\langle\psi^{(\mathrm{het})}\left(q,y\right)\right| \tag{12}$$

$$\left|\psi^{(\mathrm{het})}\left(q,y\right)\right\rangle = \left\langle 0\right|\hat{B}^{\dagger}\left|q;\hat{x}\right\rangle\left|y;\hat{p}\right\rangle = \left|\sqrt{2}\alpha\right\rangle \tag{13}$$

where $\alpha = \left(q+iy\right)/\sqrt{2\hbar}$ is a complex amplitude and $\left|\sqrt{2}\alpha\right\rangle$ is a coherent state.

In our nonlinear quadrature measurement, the heterodyne measurement is generalized by employing an adaptive nonlinear feedforward $\theta(q) = \arctan\left(\sqrt{2}\gamma q\right)$ and a non-Gaussian ancillary state. With a pure ancillary state $\left|\psi\right\rangle_{\mathrm{anc}}$, the POVM element altered by the nonlinear feedforward to a basis of the second homodyne detector is,

$$\hat{\Pi}_{q,y}^{(\mathrm{nqm})} = \frac{2}{\left|\cos\theta(q)\right|}\left|\psi^{(\mathrm{nqm})}\left(q,y\right)\right\rangle\left\langle\psi^{(\mathrm{nqm})}\left(q,y\right)\right| \tag{14}$$

$$\left|\psi^{(\mathrm{nqm})}\left(q,y\right)\right\rangle = \hat{U}(q,y)\hat{T}\left|\psi_{\mathrm{anc}}\right\rangle \tag{15}$$

$$\hat{U}(q,y) = \hat{P}\left(\tan\theta(q)\right)\hat{D}\left(\sqrt{2}q, \frac{\sqrt{2}y}{\cos\theta(q)} - \sqrt{2}q\tan\theta(q)\right) \tag{16}$$

where $\hat{P}(k) = \exp\left[ik\hat{x}^2/\hbar\right]$ is a shear operation, $\hat{D}$ is a displacement operation and $\hat{T}$ is an anti-unitary operation, which transforms $\hat{x} \to \hat{x}$ and $\hat{p} \to -\hat{p}$. The anti-unitary operator is derived from the bra of ancillary state. The details of the calculations are described in [4].

Another POVM element $\hat{\Pi}^{(\mathrm{nqm})}_{q,y'}$, which is associated with different measurement outcome y', is just displaced in the p-direction from $\hat{\Pi}^{(\mathrm{nqm})}_{q,y}$ because y in $U(q,y)$ decides only the amount of p-displacement.

When the ancillary state is an ideal cubic phase state $|\mathrm{CPS}\rangle = |0;\hat{p}-\gamma\hat{x}^2\rangle$,

$$\hat{U}(q,y)\hat{T}\,|0;\hat{p}-\gamma\hat{x}^2\rangle = \left|\frac{\sqrt{2}y}{\cos\theta(q)};\hat{p}+\gamma\hat{x}^2\right\rangle \tag{17}$$

Hence, this measurement is a projective measurement of $\hat{p}+\gamma\hat{x}^2$, with the measurement outcomes $m = \sqrt{2}y/\cos\theta(q)$. Intuitively, the shear operation recovers p-axial symmetry of a parabolic shape of the cubic phase state, which is broken by the displacement operation.

The operations $\hat{U}(q,y)\hat{T}$ transforms the nonlinear quadrature operator to a similar nonlinear quadrature operator.

$$\hat{U}(q,y)\hat{T}\left(\hat{p}+\gamma\hat{x}^2\right)\hat{T}^\dagger\hat{U}^\dagger(q,y) = -\left(\hat{p}-\gamma\hat{x}^2\right) - \frac{\sqrt{2}y}{\cos\theta(q)} \tag{18}$$

Note that the sign of the coefficient γ is flipped by the anti-unitary operation. This means that the variance of nonlinear quadrature $\hat{p}-\gamma\hat{x}^2$ of the projected state is as same as that of $\hat{p}+\gamma\hat{x}^2$ of the ancillary state. Hence, the variance of $\hat{p}+\gamma\hat{x}^2$ of a normalized POVM element $\hat{\Pi}^{(\mathrm{nqm})}_{q,y}/\mathrm{Tr}\left[\hat{\Pi}^{(\mathrm{nqm})}_{q,y}\right]$ is preserved even when the ancillary state is a mixed state. This means that the nonlinear squeezing of the ancillary state is transferred to the POVM elements by our nonlinear feedforward.

If we focus on the measurement outcomes of our nonlinear quadrature measurement, $m = \sqrt{2}y/\cos\theta(q)$, the POVM element $\hat{\Pi}_m$ is represented by a simple integration of $\hat{\Pi}^{(\mathrm{nqm})}_{q,y}$ with respect to q, because only one real y always exists for given m and arbitrary real q.

$$\hat{\Pi}_m = \int_{-\infty}^{\infty} \mathrm{d}q\,\hat{\Pi}^{(\mathrm{nqm})}_{q,\sqrt{2}m/\cos\theta(q)} \tag{19}$$

Since the POVM elements $\hat{\Pi}_{q,y}$ for all q and y preserve the nonlinear quadrature from the ancilla, even though the sign is flipped, $\hat{\Pi}_m$ also keeps the same nonlinear squeezing as $\hat{\Pi}_{q,y}$.

In actual experiment, we consider a finite interval of integral $[-r, r]$ since the range of the probe coherent states is limited by the experimental constraint.

$$\hat{\Pi}_m^{(\mathrm{exp})} = \int_{-r}^{r} \mathrm{d}q \, \hat{\Pi}^{(\mathrm{nqm})}_{q, \sqrt{2}m/\cos\theta(q)} \tag{20}$$

The parameter r is determined by the range of the amplitude of the coherent states and the POVM elements.

Supplementary Note 8 - Nonlinear quadrature measurement with experimental imperfection

In this section, we consider the model of our nonlinear quadrature measurement including experimental imperfection (optical losses and phase fluctuations). If we assume the efficiency is equivalent to linear loss, a detector state of a homodyne detection with the detection efficiency η is modeled as followed,

$$\hat{\Pi}_\eta(q|\theta) = \int |tq + ry; \hat{x}_\theta\rangle \langle tq + ry; \hat{x}_\theta| \, |\langle 0; \hat{n}| - rq + ty; \hat{x}_\theta\rangle|^2 \mathrm{d}y \tag{21}$$

$$t = \sqrt{\eta}, r = \sqrt{1-\eta} \tag{22}$$

where q is the measurement outcome and θ is the measurement basis.

With this incomplete homodyne detector, the whole setup of our adaptive measurement is modeled as,

$$\hat{\Pi}(q, y) = \mathrm{Tr}_{\mathrm{A}} \left[\hat{B} \left(\hat{\Pi}_{\eta_1}(q|0) \otimes \hat{\Pi}_{\eta_2}(y|\theta(q)) \right) \hat{B}^\dagger \hat{\rho}_{\mathrm{A}} \right] \tag{23}$$

where q, y are associated measurement outcomes, $\theta(q) = \arctan\left(\sqrt{2}\gamma q\right) + \frac{\pi}{2}$ is the rotated angle determined by the nonlinear feedforward, η_1 and η_2 are efficiencies of two homodyne detectors including propagation losses after the beam splitter, and $\hat{\rho}_{\mathrm{A}}$ is the ancillary state.

The main source of the phase fluctuation in our setup is the optical delay line. This phase fluctuation can be included in the model as the noise on feedforward, mixing the measurement

with different phases.

$$\hat{\Pi} = \int \mathrm{d}\phi\ p(\phi)\ \mathrm{Tr}_{\mathrm{A}} \left[\hat{B} \left(\hat{\Pi}_{\eta_1}(q|0) \otimes \hat{\Pi}_{\eta_2}(y|\theta(q) + \phi) \right) \hat{B}^{\dagger} \hat{\rho}_{\mathrm{A}} \right] \tag{24}$$

where $p(\phi)$ is a probability density distribution of the phase fluctuation ϕ.

Supplementary References

[1] Yuen, H. & Shapiro, J. Optical communication with two-photon coherent states–part iii: Quantum measurements realizable with photoemissive detectors. *IEEE Transactions on Information Theory* **26**, 78–92 (1980).

[2] Fiurášek, J. Maximum-likelihood estimation of quantum measurement. *Phys. Rev. A* **64**, 024102 (2001).

[3] Konno, S. *et al.* Nonlinear squeezing for measurement-based non-gaussian operations in time domain. *Phys. Rev. Applied* **15**, 024024 (2021).

[4] Miyata, K. *et al.* Implementation of a quantum cubic gate by an adaptive non-gaussian measurement. *Phys. Rev. A* **93**, 022301 (2016).
